# TEAD4, YAP1 and WWTR1 prevent the premature onset of pluripotency prior to the 16-cell stage

**DOI:** 10.1242/dev.179861

**Published:** 2019-09-01

**Authors:** Tristan Frum, Jennifer L. Watts, Amy Ralston

**Affiliations:** 1 Department of Biochemistry and Molecular Biology, Michigan State University, East Lansing, MI 48824, USA; 2 Physiology Graduate Program, Michigan State University, East Lansing, MI 48824, USA; 3 Reproductive and Developmental Biology Training Program, Michigan State University, East Lansing, MI 48824, USA

**Keywords:** Preimplantation, Stem cell progenitors, HIPPO signaling

## Abstract

In mice, pluripotent cells are thought to derive from cells buried inside the embryo around the 16-cell stage. *Sox2* is the only pluripotency gene known to be expressed specifically within inside cells at this stage. To understand how pluripotency is established, we therefore investigated the mechanisms regulating the initial activation of *Sox2* expression. Surprisingly, *Sox2* expression initiated normally in the absence of both *Nanog* and *Oct4* (*Pou5f1*), highlighting differences between embryo and stem cell models of pluripotency. However, we observed precocious ectopic expression of *Sox2* prior to the 16-cell stage in the absence of *Yap1*, *Wwtr1* and *Tead4*. Interestingly, the repression of premature *Sox2* expression was sensitive to LATS kinase activity, even though LATS proteins normally do not limit activity of TEAD4, YAP1 and WWTR1 during these early stages. Finally, we present evidence for direct transcriptional repression of *Sox2* by YAP1, WWTR1 and TEAD4*.* Taken together, our observations reveal that, while embryos are initially competent to express *Sox2* as early as the four-cell stage, transcriptional repression prevents the premature expression of *Sox2*, thereby restricting the pluripotency program to the stage when inside cells are first created.

## Abstract (precis)

**Highlighted Article:** The pluripotency marker SOX2 is not initially regulated by OCT4 and NANOG, but by HIPPO pathway members during the first 2 days of mouse embryogenesis.

## INTRODUCTION

Pluripotency describes the developmental potential to produce all adult cell types. However, in mammals, the establishment of pluripotency takes place in the context of lineage decisions that establish the extra-embryonic lineages such as the placenta and yolk sac (Chazaud and Yamanaka, 2016; Lanner, 2014; Posfai et al., 2014). The mouse embryo has provided an invaluable tool with which to understand the molecular mechanisms that initially create pluripotent cells, which are also the progenitors of embryonic stem cells. Although much progress has been made in understanding how pluripotency is maintained once pluripotent cells are established, the mechanisms driving the initial establishment of pluripotency remain relatively obscure.

In the mouse embryo, pluripotent cells emerge from the inner cell mass of the blastocyst. Establishment of inner cell mass first occurs around the 16-cell stage, when select cells occupy the inside of the morula (Posfai et al., 2014). Later, at embryonic day (E) E3.75 blastocyst stage, the inner cell mass differentiates into either pluripotent epiblast or non-pluripotent primitive endoderm (Chazaud et al., 2006; Morris et al., 2010; Plusa et al., 2008; Saiz et al., 2016; Yamanaka et al., 2010). As the epiblast matures, it gradually acquires a more embryonic stem cell-like transcriptional signature (Boroviak et al., 2014, 2015).

Although studies in mammalian embryos and embryonic stem cells have developed an extensive catalog of transcription factors that promote pluripotency, the only pluripotency-promoting transcription factor known to distinguish inside cells as they form at the 16-cell stage is *Sox2* (Guo et al., 2010; Wicklow et al., 2014). At this stage, other pluripotency factors, such as *Nanog* and *Oct4*, are detected in both inside and outside cells (Dietrich and Hiiragi, 2007; Palmieri et al., 1994; Strumpf et al., 2005). Therefore, understanding how *Sox2* expression is regulated at the 16-cell stage can provide unique insight into how pluripotency is first established.

Here, we use genetic approaches to test mechanistic models of the initial activation of *Sox2* expression. We investigate the contribution, at the 16-cell stage and earlier, of factors and pathways that are known to regulate expression of *Sox2* at later preimplantation stages and in embryonic stem cells. We show that embryos are competent to express high levels of *Sox2* as early as the four-cell stage, although they normally do not do so. Finally, we uncover the molecular mechanisms that ensure that *Sox2* expression remains repressed until the appropriate developmental stage.

## RESULTS AND DISCUSSION

### The initiation of *Sox2* expression is *Nanog-* and *Oct4*-independent

To identify mechanisms contributing to the onset of *Sox2* expression in the embryo, we first focused on the role of transcription factors that are required for *Sox2* expression in embryonic stem cells. The core pluripotency genes *Nanog* and *Oct4* (*Pou5f1*) are required for *Sox2* expression in embryonic stem cells (Chambers et al., 2003; Mitsui et al., 2003; Okumura-Nakanishi et al., 2005) and are expressed in embryos at the eight-cell stage (Dietrich and Hiiragi, 2007; Palmieri et al., 1994; Rosner et al., 1990; Strumpf et al., 2005), prior to the onset of *Sox2* expression at the 16-cell stage, raising the possibility that NANOG and OCT4 could activate the initial expression of *Sox2*.

**Fig. 1.**
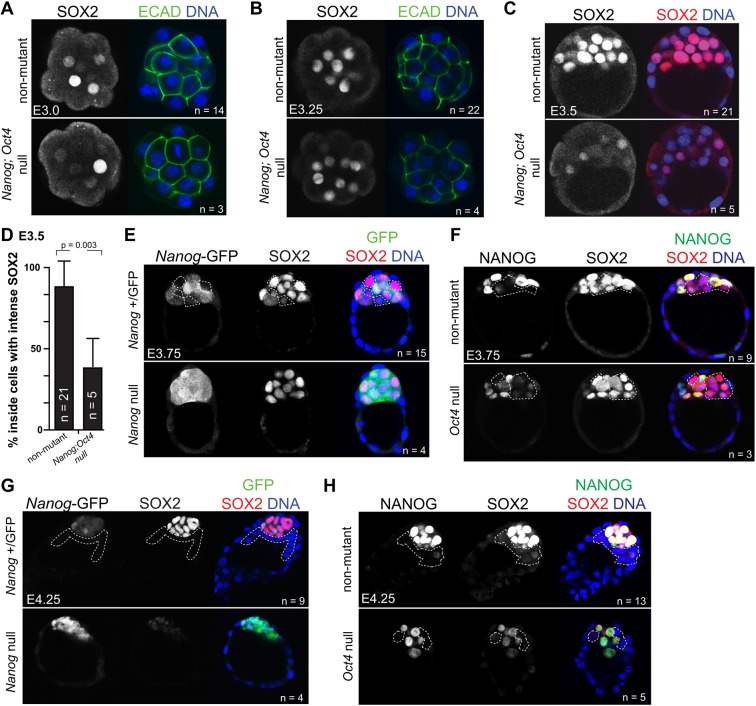
***Nanog* and *Oct4* are required for the maintenance, but not the initiation, of *Sox2*.** (A) Immunostaining for SOX2, E-cadherin (ECAD) and DNA in non-mutant and *Nanog;Oct4* null embryos at the 16-cell stage (E3.0). (B) SOX2, ECAD and DNA in non-mutant and *Nanog;Oct4* null embryos at the 32-cell stage (E3.25). (C) SOX2 and DNA in non-mutant and *Nanog;Oct4* null embryos at E3.5. (D) Manual counting of the percentage of inside cells, across all embryos, exhibiting intense SOX2 staining in non-mutant and *Nanog;Oct4* null embryos at E3.5 (see Materials and Methods for details). Data are mean±s.d., Student's *t*-test. (E) NANOG-GFP, SOX2 and DNA in non-mutant and *Nanog* null embryos at E3.75. (F) NANOG, SOX2 and DNA in non-mutant and *Oct4* null embryos at E3.75. (G) NANOG-GFP, SOX2 and DNA in non-mutant and *Nanog* null embryos at E4.25. (H) NANOG, SOX2 and DNA in non-mutant and *Oct4* null embryos at E4.25. For all panels, *n* indicates number of embryos examined. Dashed white lines demarcate non-epiblast/presumptive primitive endoderm cells.

We previously showed that the initiation of *Sox2* expression is *Oct4* independent, as normal levels of SOX2 are detected in blastocysts at E3.5 in the absence of *Oct4* (Frum et al., 2013). We therefore hypothesized that *Nanog* and *Oct4* could act redundantly to initiate *Sox2* expression. To test this hypothesis, we bred mice carrying the null allele *Nanog-GFP* (Maherali et al., 2007) with mice carrying a deleted allele of *Oct4* (Kehler et al., 2004) to generate *Nanog;Oct4* null embryos (Fig. S1A). In wild-type embryos, *Sox2* is first detected in inside cells at the 16-cell stage, with increasing levels in inside cells of the 32-cell stage embryo (Frum et al., 2013; Guo et al., 2010). In *Nanog;Oct4* null embryos, SOX2 was detectable at the 16-cell (E3.0) and 32-cell (E3.25) stages (Fig. 1A,B). We observed no difference in the proportions of SOX2-expressing cells at the 16- and 32-cell stages between non-mutant embryos and embryos lacking *Nanog* or *Oct4* or both (Fig. S1B,C), nor did we observe a difference in total cell numbers among the genotypes at any early stage examined (Fig. S1E-G). These observations indicate that *Nanog* and *Oct4* do not regulate initial *Sox2* expression.

### *Nanog* and *Oct4* are individually required to maintain *Sox2* expression

To investigate a role for *Nanog* and *Oct4* in maintaining expression of *Sox2*, we evaluated double knockout embryos at a later time point. By E3.5, SOX2 appeared weak or undetectable in most cells of *Nanog;Oct4* null embryos (Fig. 1C). Moreover, the proportion of cells expressing a wild-type level of SOX2 was significantly lower in *Nanog;Oct4* null embryos (Fig. 1D), but not in embryos lacking *Nanog* or *Oct4* only (Fig. S1D). We therefore conclude that *Nanog* and *Oct4* redundantly maintain *Sox2* expression up to E3.5.

To evaluate whether *Nanog* and *Oct4* redundantly maintain *Sox2* expression later, we examined SOX2 in embryos lacking either *Nanog* or *Oct4* at E3.75 and E4.25. At E3.75, SOX2 levels were similar among non-mutant, *Nanog* null and *Oct4* null embryos (Fig. 1E,F). Notably, *Nanog-GFP* was detected in all inner cell mass cells in the *Nanog* null embryos (Fig. 1E,G), compared with non-mutants and *Oct4* null embryos, in which NANOG was downregulated in non-epiblast cells. Therefore, *Nanog* is required for repression of *Nanog* expression in primitive endoderm. This observation is consistent with a non cell-autonomous requirement for *Nanog* in promoting primitive endoderm fate (Frankenberg et al., 2011; Messerschmidt and Kemler, 2010).

By contrast, both *Nanog* null and *Oct4* null embryos exhibited defects in SOX2 by E4.25. *Nanog* null embryos exhibited the more severe SOX2 expression phenotype, with almost undetectable SOX2 (Fig. 1G). *Oct4* null embryos exhibited a less severe SOX2 expression phenotype, with reduced, but detectable SOX2 (Fig. 1H), possibly owing to developmental delay in *Oct4* null mutants at E4.25 (Frum et al., 2013). These observations indicate that, although the initial phase of *Sox2* expression is independent of *Nanog* and *Oct4*, this is followed by a period during which *Nanog* and *Oct4* act redundantly to maintain *Sox2* expression, which then gives way to a phase during which *Nanog* and *Oct4* are individually required to achieve maximal *Sox2* expression.

### TEAD4, WWTR1 and YAP1 regulate the onset of *Sox2* expression

Having observed that the initiation of *Sox2* expression is *Nanog* and *Oct4* independent, we next examined the role of other factors in regulating initial *Sox2* expression. TEAD4 and its co-factors WWTR1 and YAP1 repress *Sox2* expression in outside cells, starting around the 16-cell stage (Frum et al., 2018; Wicklow et al., 2014). However, YAP1 is detected within nuclei as early as the four-cell stage (Nishioka et al., 2009), suggesting that the TEAD4/WWTR1/YAP1 complex is active prior to the 16-cell stage. Recent studies have highlighted the roles and regulation of TEAD4/WWTR1/YAP1 in promoting expression of CDX2 during outside cell maturation to trophectoderm during blastocyst formation (Anani et al., 2014; Cao et al., 2015; Cockburn et al., 2013; Hirate et al., 2013; Kono et al., 2014; Leung and Zernicka-Goetz, 2013; Lorthongpanich et al., 2013; Menchero et al., 2019; Nishioka et al., 2009; Posfai et al., 2017; Rayon et al., 2014; Shi et al., 2017; Yagi et al., 2007; Yu et al., 2016). Yet the developmental requirement for TEAD4/WWTR1/YAP1 prior to the 16-cell stage has not been investigated. We therefore hypothesized that TEAD4/WWTR1/YAP1 repress *Sox2* expression prior to the 16-cell stage.

**Fig. 2.**
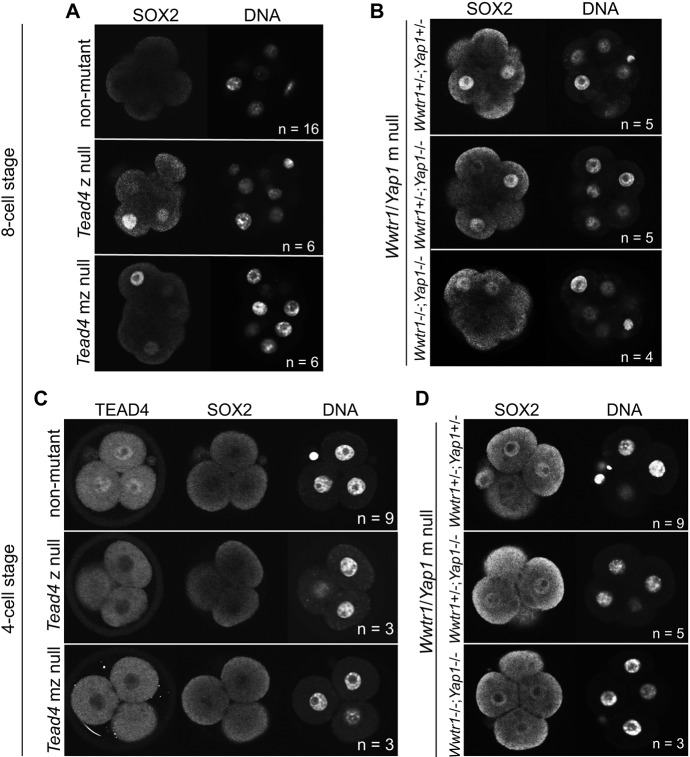
**TEAD4/WWTR1/YAP1 represses precocious *Sox2* expression at the eight-cell stage.** (A) Immunostaining for SOX2 in non-mutant, *Tead4* zygotic (z) null and *Tead4* maternal-zygotic (mz) null embryos at the eight-cell stage. (B) SOX2 in embryos lacking m *Wwtr1* and *Yap1* at the eight-cell stage, with indicated zygotic genotypes. (C) SOX2 in embryos of indicated genotypes at the four-cell stage. (D) SOX2 in embryos of indicated genotypes at the four-cell stage. *n* indicates number of embryos examined.

To test this hypothesis, we examined SOX2 in embryos lacking *Tead4*. Consistent with our hypothesis, *Tead4* null embryos exhibited precocious SOX2 at the eight-cell stage (Fig. 2A and Fig. S2C). Notably, this phenotype was not exacerbated by elimination of maternal *Tead4* (Fig. 2A and Fig. S2A,C), consistent with the absence of detectable *Tead4* in oocytes (Yagi et al., 2007). By contrast, deletion of maternal *Wwtr1* and *Yap1* (Fig. S2B) led to precocious SOX2 at the eight-cell stage (Fig. 2B and Fig. S2C). The presence of wild-type, paternal alleles of *Wwtr1* and/or *Yap1* did not rescue precocious SOX2 in the maternally null embryos. Therefore, maternally provided WWTR1/YAP1 and zygotically expressed TEAD4 repress *Sox2* expression at the eight-cell stage.

We next evaluated SOX2 in embryos lacking maternal (m) and/or zygotic (z) *Tead4* or *Wwtr1;Yap1* at the four-cell stage. We observed that SOX2 was never detected in four-cell *Tead4* z null or *Tead4* mz null embryos (Fig. 2C and Fig. S2D). However, four-cell embryos lacking maternal *Wwtr1* and *Yap1* occasionally exhibited weak ectopic SOX2 (Fig. 2D and Fig. S2D). These observations suggest that *Wwtr1* and *Yap1* partner with factors other than TEAD4 to repress *Sox2* expression at the four-cell stage. As TEAD1 and TEAD2 are also detected during the two- to eight-cell stages (Nishioka et al., 2008), we predict that these factors may partner with YAP1/WWTR1 to repress SOX2 during early embryogenesis.

The premature onset of *Sox2* expression in embryos lacking *Tead4* or *Wwtr1* and *Yap1* demonstrates that preimplantation mouse embryos are capable of expressing markers of inside cell identity as early as the four-cell stage and reveals an earlier than expected role for TEAD4/WWTR1/YAP1 in repressing the expression of *Sox2* until the formation of inside cells, thus permitting the establishment of discrete trophectoderm and inner cell mass domains of gene expression. Notably, expression of OCT4 and NANOG is unchanged in embryos lacking *Tead4* (Nishioka et al., 2008), highlighting the unique regulation of SOX2 in defining initial inner cell mass identity. Whether other pluripotency factors exist that are co-regulated with *Sox2*, remains an unresolved issue. Our results suggest that the mechanism regulating the onset of *Sox2* expression is that constitutive repression of *Sox2* by TEAD4/WWTR1/YAP1 is relieved once cells are positioned inside the embryo at the 16-cell stage. The mechanisms that initiate expression of TEAD4, WWTR1 and YAP1 prior to compaction are currently unknown.

### Repression of *Sox2* at the four- and eight-cell stages is sensitive to LATS2 kinase

In many contexts, TEAD4/WWTR1/YAP1 activity is repressed by the HIPPO pathway LATS1 and LATS2 kinases, which repress nuclear localization of WWTR1/YAP1 (Zhao et al., 2007, 2010). For example, during blastocyst formation, LATS1 and LATS2 repress nuclear localization of WWTR1/YAP1 in inside cells (Nishioka et al., 2009). To evaluate the role of the HIPPO pathway in regulating initial *Sox2* expression, we examined whether *Sox2* expression is LATS1/2-sensitive prior to the 16-cell stage.

**Fig. 3.**
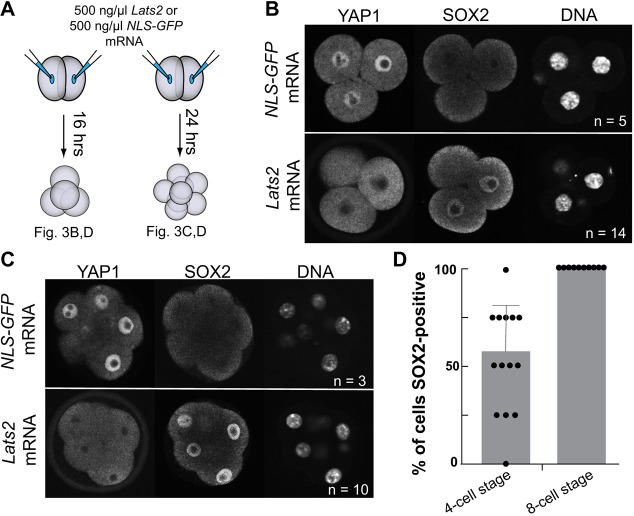
**YAP1 localization and *Sox2* expression are sensitive to LATS2 kinase.** (A) Experimental approach: both blastomeres of two-cell stage embryos were injected with either 500 ng/µl *NLS-GFP* mRNA, which encodes GFP bearing a nuclear localization sequence (NLS), or 500 ng/µl *Lats2* mRNA, and were then cultured to the four- or eight-cell stages. (B) YAP1 and SOX2 immunostaining in four-cell stage embryos injected with *NLS-GFP* mRNA or *Lats2* mRNA. (C) YAP1 and SOX2 in eight-cell stage embryos injected with *NLS-GFP* mRNA or *Lats2* mRNA. (D) The percentage of SOX2-positive cells per embryo (each value obtained is indicated by a dot) at the indicated stages. Data are mean±s.d. *n* indicates number of embryos examined.

We injected mRNA encoding *Lats2* into both blastomeres of two-cell stage embryos, which is sufficient to inactivate the TEAD4/WWTR1/YAP1 complex during blastocyst formation (Nishioka et al., 2009; Wicklow et al., 2014), and then evaluated SOX2 at the four- and eight-cell stages (Fig. 3A). As anticipated, *Lats2* mRNA injection, but not injection of green fluorescent protein (GFP) mRNA, greatly reduced YAP1 nuclear localization at four- and eight-cell stages (Fig. 3B,C). In addition, we observed precocious SOX2 in embryos overexpressing *Lats2* (Fig. 3B-D). Therefore, LATS kinases can repress TEAD4/WWTR1/YAP1 nuclear activity and induce *Sox2* expression prior to the 16-cell stage, but must not normally do so, as SOX2 is not detected prior to the 16-cell stage. After the 16-cell stage, LATS1/2 kinases are thought to be active specifically in inside cells, owing to their unpolarized state (Hirate et al., 2013; Kono et al., 2014; Leung and Zernicka-Goetz, 2013). Therefore, the polarization of all blastomeres of the eight-cell stage embryo (Frum and Ralston, 2018), or other polarity-independent mechanisms, could limit LATS1/LATS2 activation prior to the 16-cell stage.

### TEAD4/WWTR1/YAP1 may repress *Sox2* expression through a direct mechanism

**Fig. 4.**
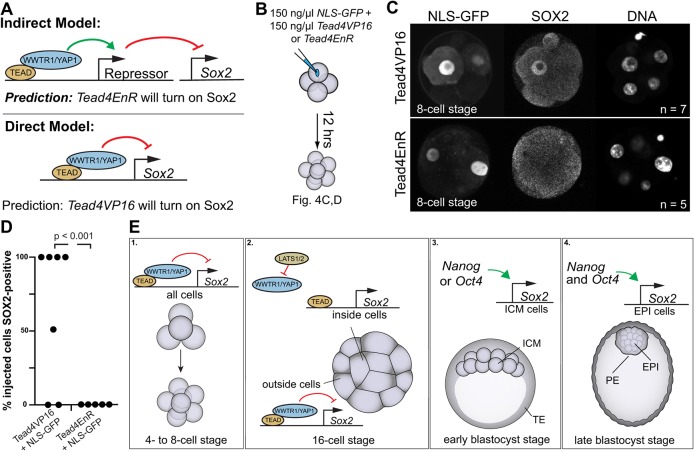
**TEAD4/WWTR1/YAP1 repress *Sox2* expression through a direct mechanism.** (A) Models for indirect and direct repression of *Sox2* by TEAD4/WWTR1/YAP1 and predicted effect of *Tead4EnR* and *Tead4VP16* on *Sox2* expression. (B) Experimental approach: a single blastomere of each four-cell embryo was injected with 150 ng/µl *NLS-GFP* mRNA and either 150 ng/µl *Tead4VP16* or *Tead4EnR* mRNA, and then cultured to the eight-cell stage. (C) GFP and SOX2 immunostaining in embryos injected with *Tead4VP16* or *Tead4EnR*. (D) The percentage of NLS-GFP-positive, SOX2-positive cells per embryo (each value obtained is indicated by a dot) injected with *Tead4VP16* or *Tead4EnR*. Student's *t*-test, *n* indicates number of embryos examined. (E) Model for regulation of *Sox2* at indicated developmental stages. ICM, inner cell mass; TE, trophectoderm; EPI, epiblast; PE, primitive endoderm.

While the TEAD4/WWTR1/YAP1 complex is widely recognized as a transcriptional activator, it has more recently been shown to act also as a transcriptional repressor (Beyer et al., 2013; Kim et al., 2015). Therefore, we considered two mechanisms by which TEAD4/WWTR1/YAP1 could repress *Sox2* expression (Fig. 4A): an indirect model, in which TEAD4/WWTR1/YAP1 promote transcription of a *Sox2* repressor; and a direct model, in which TEAD4/WWTR1/YAP1 themselves act as the *Sox2* repressor.

To test these models, we employed variants of *Tead4* in which the WWTR1/YAP1 interaction domain has been replaced with either the transcriptional activator domain of VP16 (*Tead4VP16*) or the transcriptional repressor domain of engrailed (*Tead4EnR*). These variants have previously been used in preimplantation embryos to provide evidence that TEAD4/WWTR1/YAP1 promotes *Cdx2* expression through a direct mechanism (Nishioka et al., 2009). We reasoned that if TEAD4/WWTR1/YAP1 represses *Sox2* indirectly, then overexpression of *Tead4EnR* would induce *Sox2* expression prematurely. Alternatively, if TEAD4/WWTR1/YAP1 represses *Sox2* directly, then *Tead4VP16* would induce *Sox2* expression prematurely. We injected mRNAs encoding *GFP* and either *Tead4VP16* or *Tead4EnR* into a single blastomere of four-cell stage embryos to observe the effects on SOX2 prior to the 16-cell stage (Fig. 4B). In these experiments, we commenced overexpression at the four-cell stage in order to achieve maximal expression levels of *Tead4VP16 a*nd *Tead4EnR* by the eight-cell stage. Moreover, we found that these constructs caused lethality at the two-cell stage, which did not enable us to study their effects on SOX2 expression at the eight-cell stage. We observed that overexpression of *Tead4VP16*, but not *Tead4EnR*, induced SOX2 at the eight-cell stage (Fig. 4C,D). These observations are consistent with the direct repression of *Sox2* by TEAD4/WWTR1/YAP1 prior to the 16-cell stage.

This study highlights distinct phases of *Sox2* regulation occurring during the establishment of pluripotency in mouse development. As early as the four-cell stage, blastomeres are competent to express *Sox2*, but this is overridden by TEAD/WWTR1/YAP1 (Fig. 4E, Box 1). Initiation of *Sox2* expression does not require *Nanog* and *Oct4*. Instead, LATS1/2 activity in inside cells relieves repression of TEAD4/WWTR1/YAP1 on *Sox2* at the 16-cell stage (Fig. 4E, Box 2). After blastocyst formation, the presence of either NANOG or OCT4 ensures that *Sox2* expression is maintained (Fig. 4E, Box 3)*.* Finally, as the embryo approaches implantation, *Nanog* and *Oct4* are both required to sustain *Sox2* expression (Fig. 4E, Box 4). Given that *Sox2* is detectable in preimplantation embryos of many mammalian species (Blakeley et al., 2015; Boroviak et al., 2018; Frankenberg et al., 2013; Goissis and Cibelli, 2014; Petropoulos et al., 2016), examining the functional requirements for HIPPO pathway members in the temporospatial regulation of *Sox2* in other species will provide exciting new insight into the evolution of pluripotency.

## MATERIALS AND METHODS

### Mouse strains

Animal care and husbandry was performed in accordance with the guidelines established by the Institutional Animal Care and Use Committee at Michigan State University. Wild-type embryos were generated by mating CD-1 mice (Charles River). Female mice used in this study were between 6 weeks and 6 months of age and males were used from 8 weeks to 9 months. Alleles and transgenes used in this study were maintained on a CD-1 background and include: *Nanog^tm1.1Hoch^* (Maherali et al., 2007), *Pou5f1^tm1Scho^* (Kehler et al., 2004), *Tead4^tm1Bnno^* (Yagi et al., 2007), *Yap1^tm1.1Eno^* (Xin et al., 2011), *Wwtr1^tm1.1Eno^* (Xin et al., 2013) and *Tg(Zp3-cre)93Knw* (de Vries et al., 2000). Conditional, floxed alleles were recombined to generate null alleles by breeding mice carrying conditional alleles to *Alpl^tm(cre)Nagy^* (Lomelí et al., 2000) mice.

### Embryo collection and culture

Embryos were collected from naturally mated mice by flushing dissected oviducts or uteri with M2 medium (Millipore-Sigma). All embryos were cultured in 5% CO_2_ at 37°C under ES cell grade mineral oil (Millipore-Sigma). Prior to embryo culture, KSOM medium (Millipore-Sigma) was equilibrated overnight in the embryo incubator.

### Embryo microinjection

cDNAs encoding *Lats2*, *Tead4VP16* and *Tead4EnR* (Nishioka et al., 2009) cloned into the pcDNA3.1 poly(A)83 plasmid (Yamagata et al., 2005) were linearized, and then used as a template to generate mRNAs for injection by the mMessage mMachine T7 transcription kit (Invitrogen). *NLS-GFP* mRNA was synthesized from linearized *NLS-GFP* plasmid (Ariotti et al., 2015) using the mMessage mMachine Sp6 transcription kit (Invitrogen). Prior to injection, synthesized mRNAs were cleaned and concentrated using the MEGAclear Transcription Clean-up Kit (Invitrogen). *Lats2* and *NLS-GFP* mRNAs were injected into both blastomeres of two-cell stage embryos at a concentration of 500 ng/µl. *Tead4VP16* or *Tead4EnR* mRNAs were injected into a single blastomere of four-cell stage embryos at a concentration of or 150 ng/µl each. *NLS-GFP* mRNA was included in four-cell stage injections at a concentration of 150 ng/µl to trace the progeny of the injected blastomere. All mRNAs were diluted in 10 mM Tris-HCl (pH 7.4) and 0.1 mM EDTA. Injections were performed using a Harvard Apparatus PL-100A microinjector.

### Immunofluorescence and confocal microscopy

Embryos were fixed in 4% formaldehyde (Polysciences) for 10 min, permeabilized with 0.5% Triton X-100 (Millipore-Sigma) for 30 min and blocked with 10% FBS and 0.1% Triton X-100 for at least 1 h at room temperature or longer at 4°C. Primary antibody incubation was performed at 4°C overnight using the following antibodies: goat anti-SOX2 (Neuromics, GT15098, 1:2000), rabbit anti-NANOG (Reprocell, RCAB002P-F, 1:400), mouse anti-Tead4 (Abcam, ab58310, 1:1000), mouse anti-YAP (Santa Cruz Biotechnology, sc101199, 1:200) and rat anti-ECAD (Millipore-Sigma, U3254, 1:500). Anti-SOX2, anti-TEAD4 and anti-YAP antibodies were validated by the absence of positive staining on embryos homozygous for null alleles encoding antibody target nuclei were labelled with either DRAQ5 (Cell Signaling Technology) or DAPI (Millipore-Sigma). Antibodies raised against IgG and coupled to Dylight 488, Cy3 or Alexa Fluor 647 (Jackson ImmunoResearch) were used to detect primary antibodies. Embryos were imaged on an Olympus FluoView FV1000 Confocal Laser Scanning Microscope using a 20× UPlanFLN objected (0.5 NA) and 5× digital zoom. Each embryo was imaged in entirety using 5 µm optical section thickness.

### Image analysis

Confocal sections of entire embryos, collected at 5 µm intervals, were analyzed using ImageJ (Schneider et al., 2012). Each nucleus was identified by DNA stain and then scored for the presence or absence of SOX2. In Fig. 1A,B, cells were classified as inside or outside on the basis of ECAD localization. For analysis of *Nanog;Oct4* null embryos in Fig. 1C,D and Fig. S1D, SOX2 staining intensity was categorized as intense or weak. Intense SOX2 staining was defined as the level observed in non-mutant embryos, which was uniform among inside cells. In Fig. 1 and Figs S1, S2, embryo genotypes were not known prior to analysis. In Figs 3 and 4 embryos were grouped according to injection performed, and therefore the researcher was not blind to embryo treatment.

### Embryo genotyping

For embryos at the eight-cell stage or older, DNA was extracted from fixed embryos after imaging using the Extract-N-Amp kit (Millipore-Sigma) in a total volume of 10 µl. For embryos at the four-cell stage, DNA was extracted from fixed embryos in a total volume of 5 µl. 1 µl of extracted DNA was used as template, with allele-specific primers (Table S1).

## Supplementary Material

Supplementary information
