# Supplementary material for: TEAD4, YAP1 and WWTR1 prevent the premature onset of pluripotency prior to the 16-cell stage
Source: Development. 2019 Sep 6;146(17):dev179861. doi: 10.1242/dev.179861 (PMC6765126; doi:10.1242/dev.179861)
Supplement: Supplementary information [file develop-146-179861-s1.pdf]

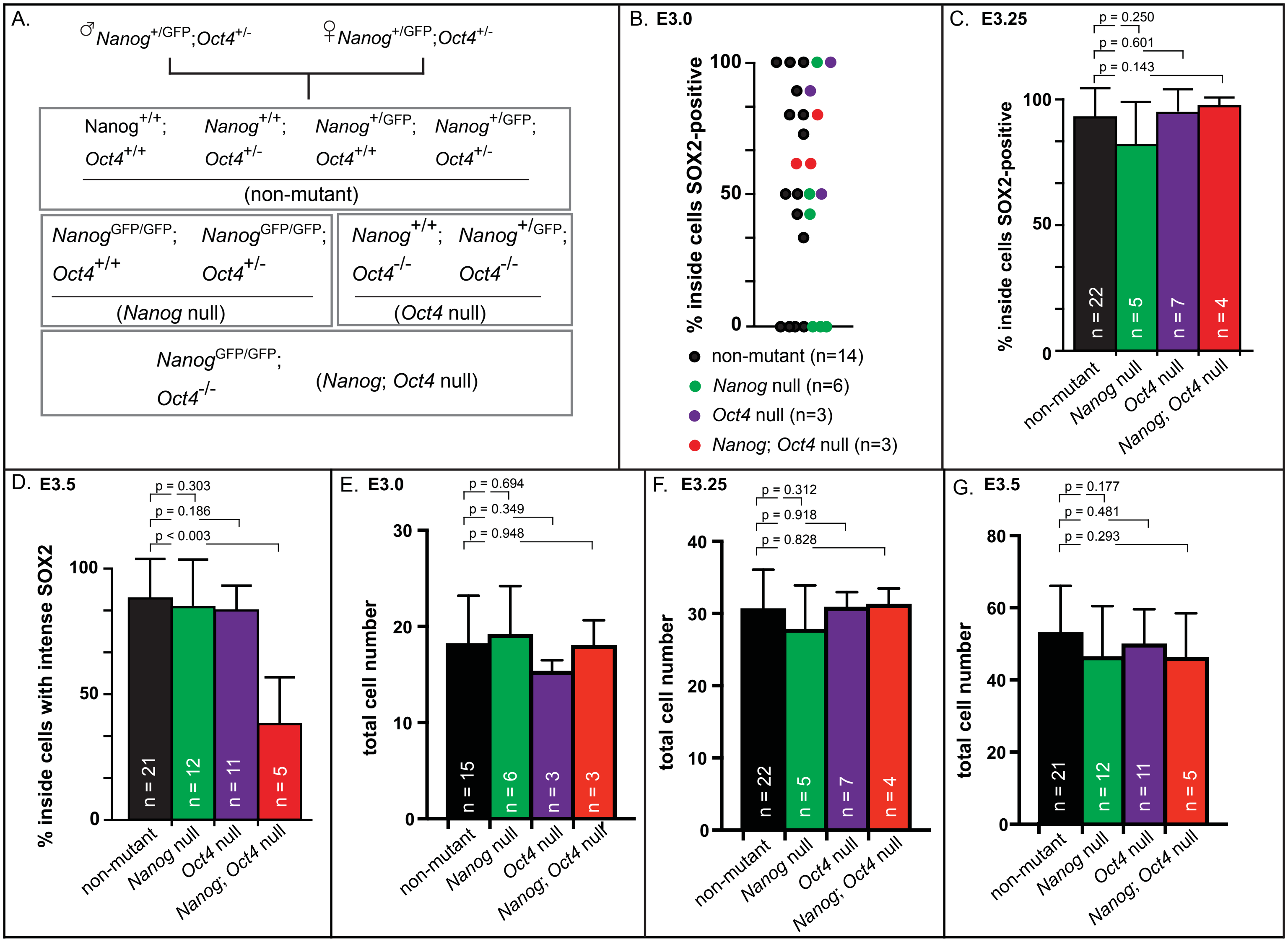

Figure S1. Breeding strategy and quantification for Figure 1. (A) Breeding strategy for generating *Nanog;Oct4* null embryos. Boxes indicate genotypes that were pooled for analysis of non-mutant; *Nanog* null (*NanogGFP*) and *Oct4* null embryos. (B) Dot-plot of the percentage of inside cells staining positive for SOX2 at E3.0 across all embryos of given genotypes. Each dot represents an embryo and is color-coded by genotype. (C) Quantification of the percentage of inside cells staining positive for SOX2 at E3.25 in genotype indicated under each column. (D) Quantification of the percentage of inside cells with intense SOX2 staining at E3.5 in embryos of indicated genotypes indicated. (E) Quantification of the total cell number for all embryos of the indicated genotype collected at E3.0. (F) Quantification of the total cell number for all embryos of the indicated genotype collected at E3.25. (G) Quantification of the total cell number for all embryos of the indicated genotype collected at E3.5. Columns = mean, error bars = standard deviation, p = Student's t-test, n = number of embryos examined.

Development • Supplementary information

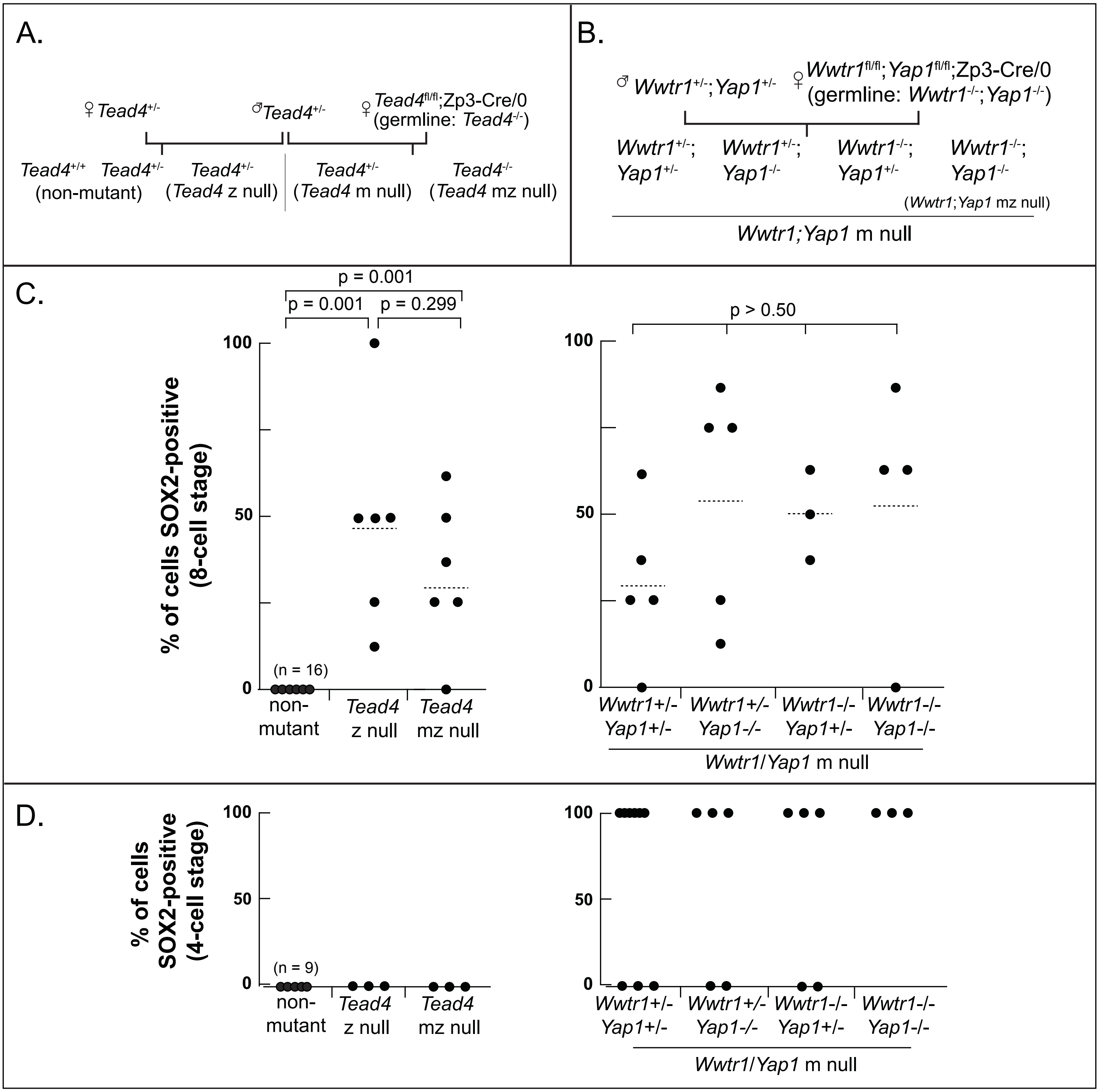

Figure S2. Breeding strategy and quantification for Figure 2. A, B) Crosses used to generate embryos examined in Figure 2. C) Proportion of total cells in which SOX2 was detected in embryos of indicated gentoypes at the 8-cell stage. D) Proportion of total cells in which SOX2 was detected in embryos of indicated gentoypes at the 4-cell stage. Dashed line = mean, p = one-way ANOVA with Tukey post-hoc test.

Development • Supplementary information

Table S1. Allele-specific primers used for determining embryo and mouse genotypes

Allele Name	Primer Name	Primer Sequence	Reference
129-Alpl$^{tm(cre)Nagy}$	CRE F	ATCCGAAAAGAAAACGTTGA	(Lomelí et al., 2000)
	CRE R	ATCCAGGTTACGGATATAGT	
Nanog$^{tm1.1Hoch}$	NANOG COMMON F	GAGAATAGGGGGTGGGTAGG	(Maherali et al., 2007)
	NANOG WT R	CCCCGAACATATTCCAAAGA	
	NANOG GFP R	CACCCCGGTGAACAGCTC	
Tg(Zp3-cre)93Knw	oIMR1084	GCGGTCTGGCAGTAAAAACTATC	(de Vries et al., 2000)
	oIMR1085	GTGAAACAGCATTGCTGTCACTT	
Wwtr1$^{tm1.1Eno}$	TAZ FL/WT F	GGCTTGTGACAAAGAACCTGGGGCTATCTGAG	(Xin et al., 2013)
	TAZ FL/WT R	CCCACAGTTAAATGCTTCTCCCAAGACTGGG	
	TAZ KO FS	TGACAAAGAACCTGGGGCTA	
	TAZ KO RS	AACTGCTAACGTCTCCTGCC	
Yap1$^{tm1.1Eno}$	YAP F	ACATGTAGGTCTGCATGCCAGAGGAGG	(Xin et al., 2011)
	YAP FL/WT R	AGGCTGAGACAGGAGGATCTCTGTGAG	
	YAP KO R	TGGTTGAGACAGCGTGCACTATGGAG	
Pou5f1^{tm1Scho}	POU5F1 FL/WT F	TTGTTACTGAAGAGGTTGGGTGTGACTGG	(Kehler et al., 2004)
	POU5F1 FL/WT R	GGGGACTCCTGCTACAACAATCGCTAAG	
	POU5F1 DEL F	AACTGGTTTGTGAGGTGTCCG	
	POU5F1 DEL R	GTATCCACTCGCACCTTGTTC	
Tead4^{tm1Bnno}	TEAD4 WT F	CTAGCATTAAGGAATGTCCCGA	(Yagi et al., 2007)
	TEAD4 WT R	CTCAACATACAGTTTGAAGCAC	
	TEAD4 FL F	CTAGCATTAAGGAATGTCCCGA	
	TEAD4 FL R	CGTATAGCATACATTATACGAAG	
	TEAD4 DEL F	CTCAACATACAGTTTGAAGCAC	
	TEAD4 DEL R	GTGTTCTTAGAGGTACAGTCA	

Development • Supplementary information
